# On the Influence of Minute Doses of Mercury, Combined with the Appropriate Treatment of Various Diseases, in Restoring the Functions of Health; and the Principles on Which It Depends

**Published:** 1834-04-01

**Authors:** 


*On the Influence of Minute Doses of Mercury, combined with the appropriate Treatment of various Diseases, in restoring the Functions of Health; and the Principles on which it depends.* By A. P. W. PHILIP, M.D., F.R.S. L. and E., &c.—London, 1834. pp. 112.

DR. WILSON PHILIP is well known to the profession as a scientific physiologist, and a distinguished practical physician: like all other mortal men, however, he has his failings, and one of them is too great a proclivity to hypothetical reasoning.

Dr. Philip has the merit of having first suggested the employment of very minute doses of mercury; and those who have taken the trouble to try the experiment cannot have failed to verify, to a considerable extent, the facts he has brought forward. Our author's view of this subject is not less consonant with reason than approved by experience. The art of medicine is always most successfully exercised when it imitates, as much as possible, the restorative efforts of nature. In acute diseases the system relieves itself by means commensurate in activity with the disease to be overcome: an affection which immediately threatens life is resolved by a rapidly increased secretion, a hemorrhage, or a sudden metastasis; while disorders of more protracted duration are overcome by a more gradual and less perceptible exertion of the sanative powers of the constitution.

The beneficial effects of mercury in various chronic diseases have been long known; but, when used in the ordinary way, the effects of the remedy are sometimes more serious than those of the disease, and it is not uncommon to witness, in the pale and emaciated victim of long-continued salivation, an impressive though not very cheering monument of the powers of the divine art. Administered, however, in the manner recommended by Dr. Philip, mercury becomes a safe as well as active remedy, in the use of which, if we occasionally fail to do good, we shall at least avoid the mortifying consciousness of having done harm.

"Large doses of mercury cannot be repeated at short intervals without often rendering the remedy as pernicious as the disease, and sometimes more so; and when they are given at distant intervals, the effect of one dose is frequently lost before another is taken; so that it often happens that little or no progress is made in the cure, and there is nothing but temporary relief to compensate for the debilitating effects of each dose; while, with respect to the minute doses, although each does little, this little it does without any strain to the constitution, and the next dose comes before the effect is lost; so that a gradual accumulation of the beneficial effect is obtained, and that, if the circumstances I am about to point out be attended to, without any injurious effects to deduct from it. The part affected is thus gradually solicited to resume its functions, and though slowly, at length effectually restored." (P. 2.)

Our author observes, that, although the utility of minute doses of mercury is most conspicuous in chronic diseases, and when its action is slow and imperceptible, still, where its more powerful and obvious effects are desired, these also may be obtained by means of the same minute doses.

"It is remarkable that, notwithstanding the general and long continued employment of mercury, it should not have been known that all its constitutional effects, not excepting complete salivation, may generally be obtained by such doses as half, or even the third part of a grain of blue-pill taken three times a day; that is a dose only equal to the twentieth or thirtieth part of a grain of calomel; for a grain of calomel is equal, whether we regard its purgative, or, when divided into minute parts, its alterative effects, to ten grains of blue pill. If such be the case, what should induce us to employ larger quantities, except the disease requires a more rapid effect than can be obtained from such doses, or, from some peculiarity in it, or the habit of the patient, the sensibility to their effects is impaired? No other person, as far as I know, has been led to the use of these doses of mercury, which, I think it will be admitted from the facts I am about to state, constitute in a great variety of cases its most beneficial employment." (P. 4.)

It sometimes happens that minute doses of mercury will excite ptyalism when large doses have failed to do so. An interesting example of this will be found in the following case:

"A lady came from a great distance to London, for the purpose, she said, of being salivated; which she had been told would relieve her from a bilious complaint, under which she had laboured for many years. For this purpose, she had taken, in vain, in the country, very large doses of mercury, much beyond the largest usually given in this climate. I saw no occasion for salivation, but directed for her, with other means, half a grain of blue pill three times a day. Her case did not require frequent visits; and not being then so well acquainted with the effects of the plan, I thought, as the mouth had resisted such doses, that no precautions respecting it were necessary, when, at one of my visits, after she had taken the medicine for about a fortnight, I found her in a state of severe salivation; the whole of the face was swelled, and she was for a considerable time confined to bed. At no great distance of time she left London well: and I learned from her sister, who two years afterwards was placed under my care, that she remained so. This lady was thus permanently cured by a quantity of mercury, the whole of which did not exceed what she had taken in vain, for a great length of time, every two or three days." (P. 35.)

Admitting the frequent utility of the small doses of mercury recommended by Dr. Philip, and the merit of that gentleman in calling the attention of the profession to the subject, we will not deny that we have experienced some disappointment from the perusal of this publication. On seeing its title, we naturally expected that the cases to which minute doses of mercury are particularly applicable would have been duly set forth, and illustrated by a sufficient number of individual examples derived from actual practice: instead of this, however, we find a large portion of the treatise occupied with matter, which, though valuable in itself, is already before the public in Dr. Philip's former works, and which is not always particularly relevant to the subject under consideration. We find also several physiological generalizations whose accuracy is extremely doubtful, and much speculation which is of no use but as an exercise of ingenuity.

The following extract from the chapter on the *modus operandi* of mercury, affords an example of the theoretical kind of reasoning alluded to above:

"I have, in my 'Inquiry into the Laws of the Vital Functions,' been at much pains to point out that there is no agent capable of affecting the living animal body that does not possess both a stimulant and sedative power with respect to it, according to the degree in which it is applied, and the state of the body at the time of its application; the stimulant arising from the less, the sedative from the greater application of it; and that the degree in which agents possess the stimulant and sedative power, although in the same agent always in the same proportion to each other, is in different agents, in no determinate, but every possible proportion.

"Thus spirit of wine possesses a great degree of stimulant compared with its sedative tendency, which only appears when it is taken in excess; while tobacco possesses a great degree of the sedative, and little stimulant tendency, which appears only when it is applied in very minute quantity.

"The sedative effect of some agents, as of opium, is chiefly exerted on the sensibility; of others, as tobacco, on the moving powers of the animal system. While the influence of the former, therefore, may be salutary, that of the latter, except under very peculiar circumstances, is always pernicious. There may be some objection to using the term sedative for agents of both descriptions. In this sense, however, it is used by writers, although not constantly, but I think it is better thus to employ it than to introduce a new term, as after this explanation no ambiguity can arise from it. Besides, as both act by diminishing the vital powers, it is convenient that there should be an appellation common to both; and what I am about to say will be sufficiently distinct, without a term to designate either alone. By sedative, then, I mean whatever depresses the powers of the system, whether sensitive or motive, and whether it affects both or either, although the more common use of the term confines it to the agents which impair the sensibility.

"No agent can impair the sensitive without more or less impairing the motive powers, because the latter in many instances depend on the former; but it is very possible to impair the motive without causing any diminution of the sensitive powers, and even with the effect of a morbid increase in them, because the derangements which accompany the weakened powers of life often prove to the sensitive powers a fruitful source of irritation. Thus, that class of sedatives whose operation is on the motive powers alone, are often doubly pernicious. Mercury, like other agents, possesses the sedative as well as the stimulant property; and its sedative property appears to be wholly exerted on the motive powers, for when it appears to lessen the sensibility, this effect seems to arise merely from its removing some cause of irritation. Its sedative tendency is very different in different constitutions; and in some it exists to a degree that wholly precludes its employment. The sedative effects of mercury, then, as of all other medicines possessing similar properties, are known by its producing a state of debility, with or without more or less nervous irritation, according to the circumstances of the particular case." (P.10.)

All this is in the very worst style of physiological reasoning: the opinion here expressed, that every substance capable of affecting the living system is, according to circumstances, a stimulant or a sedative, is one of those vague generalizations on which it is useless to argue, because it would take half a lifetime to arrange the preliminaries, and the remainder to discuss the question; with a strong probability of coming to no conclusion after all. The physiological history of the last century should for ever deter us from speculations of this nature, which can never give birth to anything but chimeras.

Our author has also hazarded some very bold and gratuitous assertions, which he does not attempt to bear out by any kind of proof: thus, when he tells us that tobacco is more injurious than opium, or indeed that it is, generally speaking, injurious at all, he lays down a position which would, we suspect, be stoutly contested by the crew of an English line-of-battle ship, who consume tobacco most exorbitantly, but who nevertheless are not usually in a very delicate state of health, and who have muscular energy enough left to floor at least six times their number of the shrivelled and mummified votaries of opium. Again, when Dr. Philip asserts, that the liver is "an organ of more intimate and extensive sympathy than the stomach," (p. 49,) we do not think he will gain many proselytes to his doctrine, which, we humbly conceive, is in the face of all observation.

The chapter on "the cases to which the minute and frequently-repeated doses of mercury are adapted, and the circumstances to be attended to in their employment," forms, on the whole, a very fair general exposition of the treatment of dyspepsia and its consequences; but, as it contains comparatively little about the minute doses of mercury, and much about divers matters which are already abundantly familiar to practitioners, we shall make no extracts from it.

The remarks "on the process by which organic disease is established" are in themselves instructive, though, like every thing else of our author's, too theoretical; but, as they consist chiefly of a condensation of some of Dr. Philip's former writings, or of very obvious inferences from them, we shall not dwell upon them here.

This treatise concludes with some observations on the employment of minute doses of mercury in *acute* diseases. The following, on protracted fever, are well worthy of attention.

"Every physician must have met with cases of fever which neither subsided as usual, nor were followed (as happens in favorable cases,) by a good appetite and a more or less rapid recovery of strength. Either the febrile symptoms continue to recur, or the patient remains languid and dispirited, and what are called the remains of the disease hang about him. In by far the majority of such cases, it will be found that more or less permanent functional disorder of the liver has been established; and although, from the chronic nature of this affection, it has not prevented the subsiding of the more urgent symptoms, it supports a constant tendency to their renewal; and, where it is not sufficient to produce this effect, it frequently prevents the recovery of the appetite, and always of the strength and spirits. The state of the liver can only with certainty be ascertained by an examination of the regions of this organ, and of the duodenum, where some tenderness or fulness will be discovered, if the cause which impedes the recovery exist in the liver, which, in consequence of the more extensive sympathies of this organ, it will be found to do in at least nineteen such cases out of twenty. Everyone will agree that, under such circumstances, all vigorous measures of a debilitating nature are out of the question; but it is not at all uncommon to see such cases aggravated by the attempt of the practitioner to restore the strength by powerful tonics, by which both the tendency to a recurrence of the fever and the oppression and restlessness are increased; and I have seen many such instances, in which the patient, guided by the effects of these means, has refused to pursue them. In the most favourable cases of this kind, they tend only to support the patient under his disease, not to relieve it; and, if their effects on the liver be not counteracted by the efforts of the constitution itself, never fail eventually to increase the mischief. The only effectual means are those which restore this organ, which is only to be here attempted by such as suit the debilitated state of the patient.

"In many instances it may be effected by a few grains of the blue-pill, taken every second night, and gently carried off by the bowels on the succeeding morning, combined with means which prevent the return of the febrile symptoms, and such stimulants as the patient can bear without any tendency of this kind, or any increase of the restlessness and oppression. In the more obstinate cases such means fail; and then I know of none which will succeed, except the substitution of the minute and frequently-repeated for the occasional larger mercurial doses, combined with the other means just mentioned, and regulated on the principles I have explained. The existence of such a case as that I am describing is, I believe, always the effect of the state of the liver having been overlooked in the course of the fever; and its frequency points out in a striking manner the necessity of attending to the state of this organ in all diseases of prolonged excitement." (P. 100.)

The disorder of the liver here adverted to is, no doubt, a frequent cause of the protraction of fever: as far as we have observed, however, quite as common a cause of such protraction consists in irritation of the mucous membrane of the bowels, kept up by the injudicious use of *purgatives*. We have seen fever kept up for days, nay, for weeks, beyond its natural duration, by purgative medicines, given for the purpose of keeping the bowels regular. Let these be relinquished in favour of laxative enemata, should such be required, and the patient will very often become rapidly convalescent.

Dr. Philip has made an allusion to the use of minute doses of mercury in chronic functional derangement of the heart, to which much attention is due. The case of Mr. Hobson is cited, which was published by that gentleman himself, in the Medical Gazette for the 22d of October, 1831.

"Mr. Hobson had for thirty-four years laboured under symptoms of diseased heart, to which all the powers of the constitution were yielding. He had become pale and œdematous, with habitually oppressed breathing, which in a great degree incapacitated him for all active duties, and rendered him subject to frequent attacks, that immediately threatened his life. Being a medical practitioner of the metropolis, he of course had the advice of many, and those the most skilful, and was regarded by all as labouring under confirmed organic disease of the heart; so that no attempt was made but with a view to present relief, and he had not for some years left his house without his name and address in his hat, fearing that he might not return alive. I was led from many circumstances, notwithstanding the severity and long continuance of the symptoms, to regard the affection of the heart as chiefly sympathetic. The function of the liver was always more or less, and occasionally much disordered; and several of the symptoms led me to believe that if organic disease of the heart did exist, it was not in sufficient extent to cause the effects 1 witnessed. For many months he steadily pursued the plan of treatment which I have laid before the reader, taking half a grain of blue-pill three times a day, combined with such means as tended to restore the digestive organs, and relieve the occasional more severe attacks. This plan had not been continued for many weeks before symptoms of amendment appeared, and in the course of a twelvemonth I had the satisfaction to see him relieved from every symptom of diseased heart. His colour became healthy, the dropsical swellings left him, and he was restored both to the appearance and functions of health." (P. 110.)

Our author adds, "I could mention several similar cases, though of less continuance; in one of which the symptoms of organic disease of the heart were quite as strongly marked, and which, after a continuance of many years, yielded as perfectly to the same means."

On the whole, we could have wished that a practical subject, like that treated of in the present publication, had been considered in a more practical manner. The truth is, that, extraneous matter apart, all that Dr. Philip had to say might have been very well communicated in a few pages of a journal, in which perhaps it might have found as appropriate a place as in a separate treatise.

